# Beyond Misconceptions: Assessing Pain in Children with Mild to Moderate Intellectual Disability

**DOI:** 10.3389/fpubh.2013.00023

**Published:** 2013-07-26

**Authors:** Marc Zabalia

**Affiliations:** ^1^Department of Psychology, Université de Caen Basse-Normandie, Caen, France

**Keywords:** pain, child, adolescent, intellectual disability, assessment

## Abstract

To assess and manage pain in children and adolescents with mild to moderate intellectual disability, healthcare providers need access to updated tools and current knowledge. Recent studies show that these children can verbally express pain and use self-assessment tools accurately. Moreover, they know pain coping strategies. Finally, they show mental imaging skills and are able to recall autobiographical memories. These new data suggest that such children and adolescents could be candidates to for hypno-analgesia protocols and behavioral relaxation.

## Introduction

People with intellectual disabilities are now recognized as a population at risk for unmanaged pain (1), after being sometimes considered insensitive to pain (2). Literature shows that this population commonly experiences pain, but it is rarely treated (3), although the prevalence of health problems is significantly higher than in the general population (4). The lack of communication impedes pain detection (5). People with intellectual disabilities are subject to the same pain as the general population, and in addition they must cope with specific disability-related pain (1, 6, 7).

Appropriate management of pain requires appropriate assessment. Caregivers need access to reliable and scientifically validated scales as well as updated knowledge about pain in this population.

Research has been slow to develop specific assessment tools, for a review, see (2, 8). Now we have validated scales for assessing chronic and acute pain in people with severe disabilities: the Pediatric Pain Profile (9) and the Non-Communicating Children Pain Checklist-Revised [NCCPC-R (10)]. However studies on the assessment of pain in children and adolescents with mild to moderate intellectual disability are still rare.

## Pain Self-Report and Intellectual Disability

Pain is a highly subjective phenomenon, self-report is appropriate in many clinical settings and it is fast and easy to use. Nevertheless in children with developmental or intellectual disabilities, self-assessment seems systematically excluded *a priori* from assessment practices.

The first study about pain self-assessment abilities in children with intellectual disabilities included 47 children with various levels of disability and 111 children without any disability, all with planned surgery (11). The aim was to assess how children used a numerical scale from 0 to 5 to rate pain intensity. Children’s understanding of the concepts of proportion and ordinal position was tested using a classification task of various sized blocks and a numbers ranking task. Then, the children were asked to sort schematic faces expressing different levels of pain intensity. Only 10 children (21%) with intellectual disabilities completed the 3 pre-test tasks, all of them with mild impairment. Twenty-three children (44%) were able to complete some of the tasks. But none of these children completed the final task of assigning numbers to the faces representing pain intensity. In the control group, all the children over 8 years old completed all the tasks, 18% of children from 4 to 7 years old completed all tests, and 32% succeeded in some tasks. These results are not surprising. The tasks proposed involve cognitive processes such as logic of classes and order relations that children with typical development can only understand near 7 or 8 years old. By definition, the vast majority of children with intellectual disabilities do not achieve this cognitive level.

Moreover, this approach perpetuates a widespread confusion in the literature about pain self-assessment in children. There is confusion between pain as an intellectualized concept and pain as a subjective sensory and emotional experience (12). Of course, in clinical settings it is the latter that the child must communicate to the adult. Pain expression and emotional expression in general, emerge from relationships with the family and other social environments, kindergarten for example (13). When assessing a phenomenon such as pain, the child probably makes an overall assessment of the “quantity of pain” (intensity) rather than mobilize order relations, because it is adaptive enough. Infants and many animals are able to roughly compare orders of proportion [for a review, see (14)].

In another study, Benini and colleagues tested self-assessment skills in 16 children aged 7–18 years with mild to moderate intellectual disability (15). In this study, children were given 1-h training on the use of assessment tools before a venipuncture. Then the children completed the original and an adapted version of the 10 cm visual analog pain scale, the Eland color scale, which shows the image of a whole body, and a six faces pain scale (16). The results showed no differences in children’s ability to use scales based on the level of impairment (mild or moderate) or etiology (Cerebral Palsy or Down syndrome). The authors report that children used simplified scales better [faces number reduced to four, parts of body enlarged in Eland scale, set of five cubes rather than visual analog scale (VAS)], but the consistency between parents, the experimenter (using a VAS), and child ratings was moderate.

This study has two major weaknesses. The first is related to the use of modified tools by the experimenters. The psychometric properties of an assessment tool cannot be guaranteed if this tool is modified in its structure or if the conditions of its use are not standardized. The second problem lies in the use of a VAS by parents and experimenters. This scale is currently only validated for self-assessment. The measure may not be valid when the tool is used to estimate pain intensity in others. Only one study has shown that the VAS is a reliable hetero-assessment (17) but it involved 12 months old infants observed during a subcutaneous injection.

Two other studies have tested pain assessment abilities in children with cognitive impairment. Zabalia et al. (18) studied the ability to use self-assessment tools in 14 children aged 8–18 years with mild to moderate intellectual disabilities (IQ from 45 to 70 assessed with the Wechsler Intelligence Scale for Children – WISC-III). Using the VAS and the Faces Pain Scale-Revised [FPS-R (19)], children were asked to assess the pain of a character in colored pictures. The pictures showed domestic burning, fall, and injection. After each presentation, the children were asked about pain felt in a similar situation and had to assess its intensity with VAS and FPS-R. This interview focuses on “referred pain” with the method of an explaining interview. This is an assisted verbalization referring to a real situation that has already occurred. Finally, children were asked to describe the nature of the pain in each situation.

Children in the study demonstrated abilities to communicate and assess pain. They used self-assessment tools to estimate the pain of a character or self-reported pain. They also used these tools more accurately when they assessed pain that they personally experienced. In this situation, the children were less likely to use only 0 or 10 ratings. The children were able to say six words on average to describe the nature of the pain, which is equal to the lexicon of typical children with the same mental age (20). The words were appropriate to the context and nature of pain.

A study explored the ability to recognize emotions and pain assessment in children and adolescents with Down syndrome (21). Down syndrome is rarely found in pain research, although it is the most common congenital etiology of intellectual disabilities in the world. The children performed an assessment of the pain of characters in pictures and an emotion recognition task. Eighteen children and adolescents with Down syndrome were compared to 20 typical children. The results showed that the children were able to identify emotions and displayed error patterns analogous to the typical population. Children and adolescents with Down syndrome used the FPS-R more appropriately than the VAS. Indeed, there is only 25% of only 0 or 10 ratings with the FPS-R (similarly to the control group) while they account for almost half of the ratings with the VAS. A limitation of these studies is that the children did not assess current pain. Ongoing work now involves having children use self-report scales during painful treatments. They will allow us to test the strength of the previous results. We must keep in mind that the main objective here is not to get from children a complete description of their painful experience but to have sufficiently stable and accurate information to make decisions about treatment. Although all aspects of painful phenomena are important to assess, the parameter most commonly used is pain intensity, as indicated by the recommendations of the Pediatric Initiative on Methods, Measurement, and Pain Assessment in Clinical Trials Consensus Group [Ped-IMMPACT (22)]. For most professionals, the distinction between no pain, mild pain, and moderate to severe pain is good enough for everyday practice (2). Getting the child’s self-report should be one of multiple ways that pain should be assessed. Parent input and healthcare practitioner assessment should also be considered to provide a comprehensive assessment in the treatment of the child. There is good evidence that although children may be able to assess their own pain, when they are actually experiencing pain their cognitive function and regular abilities are reduced (15, 23).

## Pain Coping Skills and Intellectual Disabilities

Coping skills are stress and pain adjustment, adaptation, or confrontation processes (24). These cognitive and/or behavioral responses are more or less adaptive in their results in terms of relief, emotional adjustment, or functional status of the person.

Coping is a process, it is not an intrinsic ability. In common painful experiences or during hospitalization, people develop various strategies to cope with stress and pain. Coping strategies are not innate responses. Newborns and infants do not have the resources to cope with pain. Adults are those who identify and manage a very young child’s pain. Therefore, reporting and coping with pain systematically occur in the context of an interaction. In these experiences, the child can learn particular pain words and behaviors from adults around him and gradually learn to regulate their responses independently (25).

Specific strategies have been highlighted by research in typical children’s coping skills. These studies suggested that there are passive and active strategies to cope with pain. Active strategies, such as distraction, are considered optimal and most effective to decrease the painful sensation. Passive strategies such as avoidance or seeking social support, are considered less relevant because they do not deal directly with the source of pain (26–28). Three studies focused on pain coping strategies in children and adolescents with intellectual disabilities. With the French version of the Pediatric Pain and Coping Inventory (PPCI) developed by Varni and colleagues (24) and adapted by Spicher (29), Zabalia and Duchaux (30) categorized responses from 23 children and adolescents 7–14 years old with mild to moderate intellectual disabilities (IQ from 36 to 77 calculated using the WISC-III). Children in this study were given the Pain Assessment Instrument for Cerebral Palsy [PAICP (31)]. The tool consists of eight drawings of everyday situations usually painless (brushing teeth, listening to music) and six drawings of situations usually painful (to bite his tongue, getting stung by a bee). Four test drawings are used to explain the instructions and to ensure that children understand the questionnaire. In each situation, the child must assess pain experienced by the character (whose faces are not visible) using the FPS-R (19). The authors showed that children with intellectual disabilities choose more frequently the strategy “seeking social support” as a way to cope with pain. This strategy is not considered as optimal in typical conditions but with an intellectual disability, it is probably the most efficient mechanism because these children may not have autonomous responses to pain. However, the use of this strategy may be a problem because it can be understood to be a less adaptive way to deal with the pain (32) rather than as an appropriate strategy. In a survey study about strategies used by children with intellectual disabilities, 78 parents or step-parents reported different methods their children used to cope with pain (33). Children and adolescents most often used “problem solving,” “seeking social support,” and “catastrophizing.” The authors considered that self-distraction and cognitive strategies may have exceed the child’s resources. Thus, children could be better supported if their relatives helped them use or adapt the coping strategies already within their repertoire. Another qualitative study interviewed parents of 12 children with Down syndrome aged 6–12 years (34). Although parents recognized their children to be less verbally expressive, they said their children search verbal contact and try to be close to the parent as evidence of pain.

The next section discusses how coping patterns develop in typical versus children with intellectual disabilities. Some studies focused on the development of coping strategies in adolescents (35, 36). Growing up, children with typical development rely less on social support and more on cognitive self-instruction (24). To cope with a stressful event, typical children gradually use more problem solving-focused strategies and cognitive strategies than emotion-focused strategies (37–39). The 15th year seems to be the turning point in this transition (40, 41).

In their study, Zabalia and colleagues (25) compared 28 adolescents aged 13–17 years with mild to moderate intellectual disabilities (IQ from 45 through 70, mental age from 4 through 12 years old) with 28 typical adolescents matched in gender and chronological age. In a semi-structured interview, the participants discussed their knowledge about pain coping strategies. Results indicate that adolescents with developmental disabilities expressed pain and reported coping strategies appropriately. However, having an intellectual disability seemed to limit the diversity of strategies mentioned spontaneously. The strategies presented were mainly focused on the problem (Problem solving and Seeking Social Support). The increased use of the problem solving strategy indicates that the adolescents with intellectual disabilities understood the painful phenomenon and that drugs or behaviors (rubbing the painful area for example) would have an analgesic effect. The use of the social support strategy may be an appropriate response because individuals with intellectual disabilities may lack the adaptive capacity to deal with the pain on their own. These problem focused strategies are likely to reduce anxiety when the situation is controllable, but may amplify it otherwise. Overall, adolescents with intellectual disabilities suggested appropriately their need to be with a person capable of controlling the situation.

Emotion-focused strategies are very rarely raised, as they involve mobilization of cognitive processes (cognitive self-instruction), or emotional regulation control (distraction). These strategies reduce the anxiety caused by pain when the event is uncontrollable (42). But if adolescents with intellectual disabilities rarely use this strategy, it may have important implications for iatrogenic pain. Procedural pains are uncontrollable painful events for the patient. Individual resources of children and adolescents with intellectual disabilities are probably not sufficient to mobilize a strategy of distraction on their own for example. The presence of a relative seems necessary for an appropriate strategy. One study investigated the capacity of children and adolescents with mild intellectual disability to benefit from hypno-analgesia protocols (43). Most techniques used with 6- to 12-year-old children (think of a favorite place, think of a hobby, listen to a story) involve mental imagery and autobiographical memory. Twenty-one adolescents with intellectual disabilities aged 13–20 years (mental age 3.6–9.6 years) performed a mental imagery task and an autobiographical memory task. Results indicated that adolescents were able to generate mental images when reading a story. These images appeared sufficiently vivid to be used when selecting a picture of the end of the story. However, the story should be simple and without ambiguity or unrealistic events. Assessment of autobiographical memory shows that this type of memory can be used in this population. Compared to the control group, adolescents with intellectual disabilities are capable of evoking specific personal events. Hypno-analgesia should be considered accessible to people with intellectual disabilities who have a mental age >4 years, as it is in young typically developing children. In children, self-hypnosis can reduce abdominal pain (44, 45) and headache (46, 47). Guided imagery has been used for postoperative pain in typically developing children over the age of 7 years (48), and recurrent abdominal pain over the age of 5 years (49). Training is necessary for children with intellectual disabilities; however, those with only mild levels of disability may be able to learn scripts that they could use independently afterward (1).

## Conclusion

Healthcare providers still seem to have prejudices about the relevance and efficacy of pain treatments in people with intellectual disabilities (50). Even when the pain is acknowledged, this population receives fewer analgesics than the general population (51, 52). Pain is a subjective and complex phenomenon; self-assessment may be the best defense against misconceptions. Children and adolescents with mild to moderate intellectual disability are able to assess and deal with pain. Most experts in the area suggest that almost all treatments available for pain relief can be used with people with intellectual disabilities, provided that the treatment is compatible with their mental and physical characteristics and the other treatments they are using for their pain. Multidisciplinary care is highly recommended to increase the synergistic effects of several treatment options (pharmacological and non-pharmacological). Pain assessment and treatment in this population should be a priority. Pain should no longer be an obstacle to the development of their full potential.

## Conflict of Interest Statement

The authors declare that the research was conducted in the absence of any commercial or financial relationships that could be construed as a potential conflict of interest.
